# First-in-class topical therapeutic omilancor ameliorates disease severity and inflammation through activation of LANCL2 pathway in psoriasis

**DOI:** 10.1038/s41598-021-99349-y

**Published:** 2021-10-06

**Authors:** Nuria Tubau-Juni, Raquel Hontecillas, Andrew Leber, Panita Maturavongsadit, Jyoti Chauhan, Josep Bassaganya-Riera

**Affiliations:** Landos Biopharma, Inc., Blacksburg, VA 24060 USA

**Keywords:** Drug discovery, Immunology

## Abstract

Psoriasis (PsO) is a complex immune-mediated disease that afflicts 100 million people. Omilancor is a locally-acting, small molecule that selectively activates the Lanthionine Synthetase C-like 2 (LANCL2) pathway, resulting in immunoregulatory effects at the intersection of immunity and metabolism. Topical omilancor treatment in an imiquimod-induced mouse model of PsO ameliorates disease severity, epidermal hyperplasia and acanthosis. Further, pharmacological activation of LANCL2 results in significant downregulation of proinflammatory markers including local reduction of IL17, and infiltration of proinflammatory cell subsets. These therapeutic effects were further validated in an IL-23 PsO model. This model reported increased preservation of homeostatic skin structure, accompanied by a decreased infiltration of proinflammatory T cell subsets. In CD4+ T cells and Th17 cells, the LANCL2 pathway regulates proinflammatory cytokine production, proliferation and glucose metabolism. Metabolically, the loss of Lancl2 resulted in increased glycolytic rates, lactate production and upregulated enzymatic activity of hexokinase and lactate dehydrogenase (LDH). Inhibition of LDH activity abrogated the increased proliferation rate in Lancl2^−/−^ CD4+ T cells. Additionally, topical omilancor treatment decreased the metabolic upregulation in keratinocytes, keratinocyte hyperproliferation and expression of inflammatory markers. Omilancor is a promising topical, LANCL2-targeting therapeutic candidate for the treatment of PsO and other dermatology indications.

## Introduction

Psoriasis (PsO) is one of the most frequent human skin disorders, affecting over 7 million people only in the United States^1,2^, and 100 million people worldwide, according to the WHO. PsO is a chronic autoimmune condition characterized by keratinocyte hyperproliferation and altered maturation, dermal thickening, angiogenesis and increased skin infiltration of immune cells that is presented as scaly and thickened skin plaques^3–5^. The resulting itchiness from the skin plaques, effects on appearance, scaling, and skin pain, have a significant impact on the quality of lives of patients, that report physical, social and emotional burden^6–8^. PsO is not only a skin disease, but it is also associated to the development of several conditions, including psoriatic arthritis, cardiovascular disease, type-2-diabetes, obesity and depression^9–13^. Although biological treatments are moderately successful in the treatment of PsO, long-term safety and tolerability concerns, non-responsiveness to the treatment or loss of effectiveness, and elevated cost, are the most common reasons that trigger treatment discontinuation, or even not initiation of treatment in PsO patients^14^. Thus, the unmet clinical need to develop safer and more effective drugs for PsO that will also facilitate adherence to treatment and maintain an effective long-term management of the disease is significant and must be urgently addressed.

PsO is a highly complex disorder with a multifactorial etiology, that involves genetic, environmental, lifestyle and infection factors^15–18^. Even though historically it was considered a solely skin disease, it is now recognized as a largely T cell-mediated disorder^19^. Several immune signaling pathways and cytokines are involved in the pathogenesis of PsO^20^, however, the IL23/Th17 axis has been established as the major player of this process^21,22^. Indeed, psoriatic lesional skin present upregulated levels of IL-23, IL-17, IL-22, cytokines, as well as IFNγ^23,24^. Further, Th17 cells accumulate in dermal tissue of psoriatic patients, and IL-17 expression correlates with disease activity^23^. Additionally, Treg cells from PsO patients present altered suppressive function^25^, associated to reduced Foxp3 expression, and increased disposition to differentiation into IL-17-producing cells^26^. During PsO pathogenesis, skin injury-induced cell death and keratinocyte-release of antimicrobial peptides, as well as other internal stimuli that dysregulate skin homeostasis, lead to the activation of dermal dendritic cells (DC)^27^. Then, local DC initiate production of IL-23, activating Th17 cells, and other IL-17-producing cells, that initiate secretion of several cytokines, including IL-17A, IL-17F, and IL-22^21,28^. IL-17A, IL-22, among other proinflammatory cytokines, upregulate keratinocyte proliferation, suppress keratinocyte differentiation and induce expression of chemokines and antimicrobial peptides, that feed into the inflammatory cascade, resulting in formation of plaque psoriasis with increased infiltration of neutrophils, monocytes, DC and T cells^3,29–31^. Therefore, the pathogenesis of PsO displays a significant immune cell-driven development with a substantial keratinocyte component.

Omilancor is a locally-acting, first-in-class, small molecule therapeutic, and the lead agonist of Lanthionine Synthetase C-like 2 (LANCL2)-targeting, a novel drug target for autoimmune^32–35^, metabolic^36–38^ and infectious^39,40^ diseases. LANCL2 is a G-coupled immunometabolic receptor, downstream associated to adenylate cyclase activation and calcium signaling^32,41–44^, involved in the regulation of T-cell mediated responses^34,35^. Activation of the LANCL2 pathway in the gut through oral omilancor treatment has demonstrated therapeutic efficacy in 6 different preclinical models of inflammatory bowel disease (IBD). Indeed, oral treatment with omilancor ameliorates disease severity, suppresses inflammation and enhances Treg function through immunometabolic mechanisms^34,35^. Omilancor has three open INDs (ulcerative colitis, Crohn’s disease and eosinophilic esophagitis) and is currently in clinical development for IBD with recent Phase 2 results indicating an induction of clinical remission in 31.8% of patients with ulcerative colitis.

Activation of the LANCL2 pathway in immune cells impacts cell differentiation by stabilizing regulatory phenotypes leading to downregulated expression of metabolic pathways^35^. Through LANCL2 activation, omilancor regulates late-stage glycolysis and promotes oxidative metabolism in regulatory T cells (Treg). Additionally, omilancor also modulates Treg stability and function through a two-layer mechanism: (1) regulation of FOXP3 activity through immunometabolic pathways^35^; and (2) synergism with the IL-2/STAT5 signaling axis of Treg cells^34^. Through these mechanisms, the LANCL2 pathway promotes induction of a stable Treg cell phenotype with enhanced suppressive capacity, providing therapeutic efficacy in autoimmune disease^34,35^. In addition to the therapeutic efficacy displayed by oral omilancor treatment in autoimmune diseases, the therapeutic development of omilancor is supported by a strong benign safety profile. No dose-limiting toxicities, biochemical or hematological changes, or macroscopic/microscopic changes to organs were reported upon oral omilancor treatment up to 1000 mg/kg/day orally in rats and dogs for 3 months^45^. Additionally, in a randomized, double-blind, placebo-controlled Phase I clinical trial in humans, omilancor was also well tolerated with no dose-limiting toxicities over 7000 mg/day for 7 days^46^. The benign safety profile of omilancor was also supported by the data of the Phase 2 clinical trial, reporting no emergent trends in adverse event profile in omilancor-treated patients in comparison to the placebo.

Given the role of T cell-mediated responses in the pathogenesis of PsO, implication of the LANCL2 pathway in regulating CD4+ T cell differentiation and function, and the reported efficacy and safety of omilancor in the treatment of complex autoimmune diseases such as IBD, we developed a topical formulation of omilancor to assess the therapeutic potential of topical pharmacological activation of LANCL2 in PsO. This manuscript provides evidence of therapeutic efficacy of omilancor’s topical formulation in two models of PsO, through immunometabolic down-modulation of Th17 cells.

## Results

### Lancl2 activation by topical omilancor reduces disease severity in a mouse model of imiquimod (IMQ)-induced PsO

**Figure 1:**
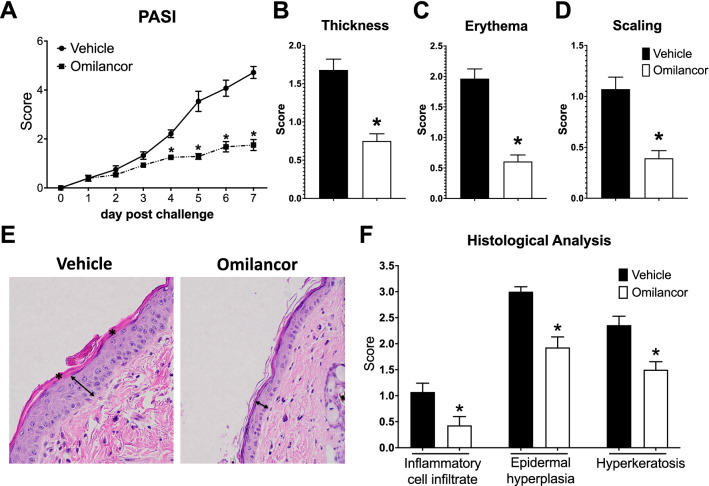
Therapeutic efficacy of topical omilancor treatment in an IMQ-induced model of PsO in dorsal skin. WT mice were shaved and challenged with IMQ treatment for 7 days. Vehicle or omilancor topical formulation was applied daily 1 h post IMQ challenge. Disease activity was monitored daily using a modified PASI (**A**). Skin thickness (**B**), erythema (**C**) and scaling (**D**) at day 7 post challenge. Representative photomicrographs of hematoxylin and eosin (H&E)-stained dorsal skin at day 7 (**E**). Epidermal hyperplasia (↔) and hyperkeratosis (*) are represented. Averaged histopathological scores for inflammatory cell infiltrate, epidermal hyperplasia and hyperkeratosis (**F**). *P ≤ 0.05.

To initially investigate the potential therapeutic efficacy of topical pharmacological activation of LANCL2 pathway during PsO, we utilized the murine IMQ-induced model, that is extensively used in PsO research. IMQ is a TLR7/8 agonist and strong immune activator that induces a potent PsO-like skin inflammation that resembles human PsO^47,48^. Wild-type (WT) mice were anesthetized, dorsal skin shaved, and briefly exposed to depilatory cream. After a 3-day resting period, mice were daily challenged with 3.125 mg of IMQ for 7 days. Mice were also treated with a topical formulation of 2% omilancor (1 mg/day omilancor), or vehicle, daily, 1 h post IMQ challenge. Topical omilancor treatment ameliorated disease severity starting on day 4, resulting in a 60% reduction of PASI score by the end of the challenge (Fig. 1A). On day 7, omilancor-treated mice displayed a twofold decrease in all three parameters analyzed, skin thickness (Fig. 1B), erythema (Fig. 1C) and scaling (Fig. 1D). At the histological level, topical omilancor reduced the appearance of typical signs of psoriatic skin (Fig. 1E). Indeed, skin sections from treated mice presented a decrease in inflammatory cell infiltrate, epidermal hyperplasia, and hyperkeratosis (Fig. 1F).

### Systemic inflammation and local recruitment of proinflammatory subsets are decreased upon omilancor topical treatment during IMQ challenge

**Figure 2:**
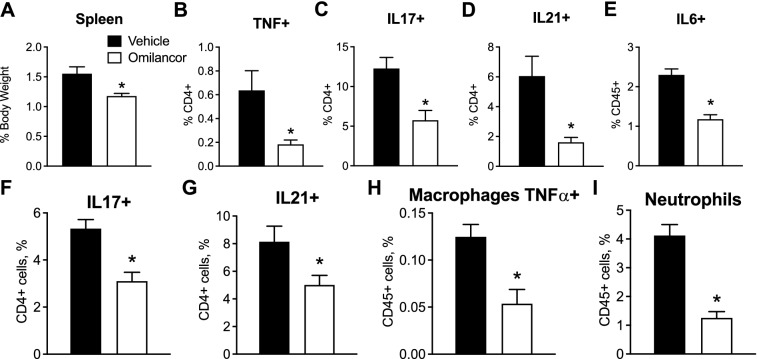
Effects of topical omilancor treatment in spleen and local draining lymph nodes (ILN) in IMQ-challenge. At day 7 post-IMQ challenge, spleen and ILN from vehicle and omilancor-treated mice were excised and single cell suspension isolated. Spleen size (**A**), represented as proportion of total body weight. Using flow cytometry, percentage of TNF+ (**B**), IL-17+ (**C**) and IL-21+ (**D**) CD4+ T cells, and IL-6+ (**E**) CD45+ cells was assessed in spleen. In draining ILNs, IL-17+ (**F**) and IL-21+ (**G**) CD4+ T cells, as well as TNFα-producing macrophages (**H**) and neutrophils (**I**) were also quantified using flow cytometry analysis. *P ≤ 0.05.

To assess the effects of omilancor treatment at the immunological level, spleen and draining lymph nodes (inguinal lymph nodes, ILN) from WT mice challenged with IMQ for 7 days, and treated with vehicle or omilancor topically, were collected and processed for flow cytometry analysis. Our results indicate that topical omilancor treatment results in a decrease in systemic inflammation, observed as a reduction in spleen size (Fig. 2A), as well as downregulation of several pro-inflammatory cytokines, including TNF (Fig. 2B), IL17 (Fig. 2C), IL21 (Fig. 2D) and IL6 (Fig. 2E). At the local level, topical omilancor treatment was associated to decreased recruitment of proinflammatory immune subsets. Isolated ILN from treated mice presented a reduced proportion of IL17-producing CD4+ T cells (Fig. 2F), IL21-producing CD4+ T cells (Fig. 2G), TNFα-producing macrophages (Fig. 2H), and neutrophils (Fig. 2I). The beneficial effects of topical omilancor treatment were validated in a pilot IMQ-induced model on mouse ear skin. WT mice were challenged with IMQ in their frontal and dorsal ear skin, followed by topical omilancor or vehicle administration, for 7 days. Topical omilancor treatment significantly reduced the overall PASI score starting at day 2 post challenge, reporting a twofold decrease of skin thickness and scaling at day 7 (Supplementary Fig. S1A,B). At the immunological level, we also observed the decrease in spleen sizes (Supplementary Fig. S1C), reported in the regular dorsal skin IMQ-induced model, as well as a slightly increase of regulatory T cells (Supplementary Fig. S1D) and a significant decrease of neutrophils (Supplementary Fig. S1E) in draining cervical lymph nodes. Thus, these results indicate that topical omilancor treatment displays therapeutic efficacy in two mouse models of IMQ-induced PsO, through suppression of both systemic and local inflammation.

### Topical treatment with omilancor modulates keratinocyte function and suppresses recruitment of effector T cell subsets upon activation of IL23/Th17 axis

**Figure 3:**
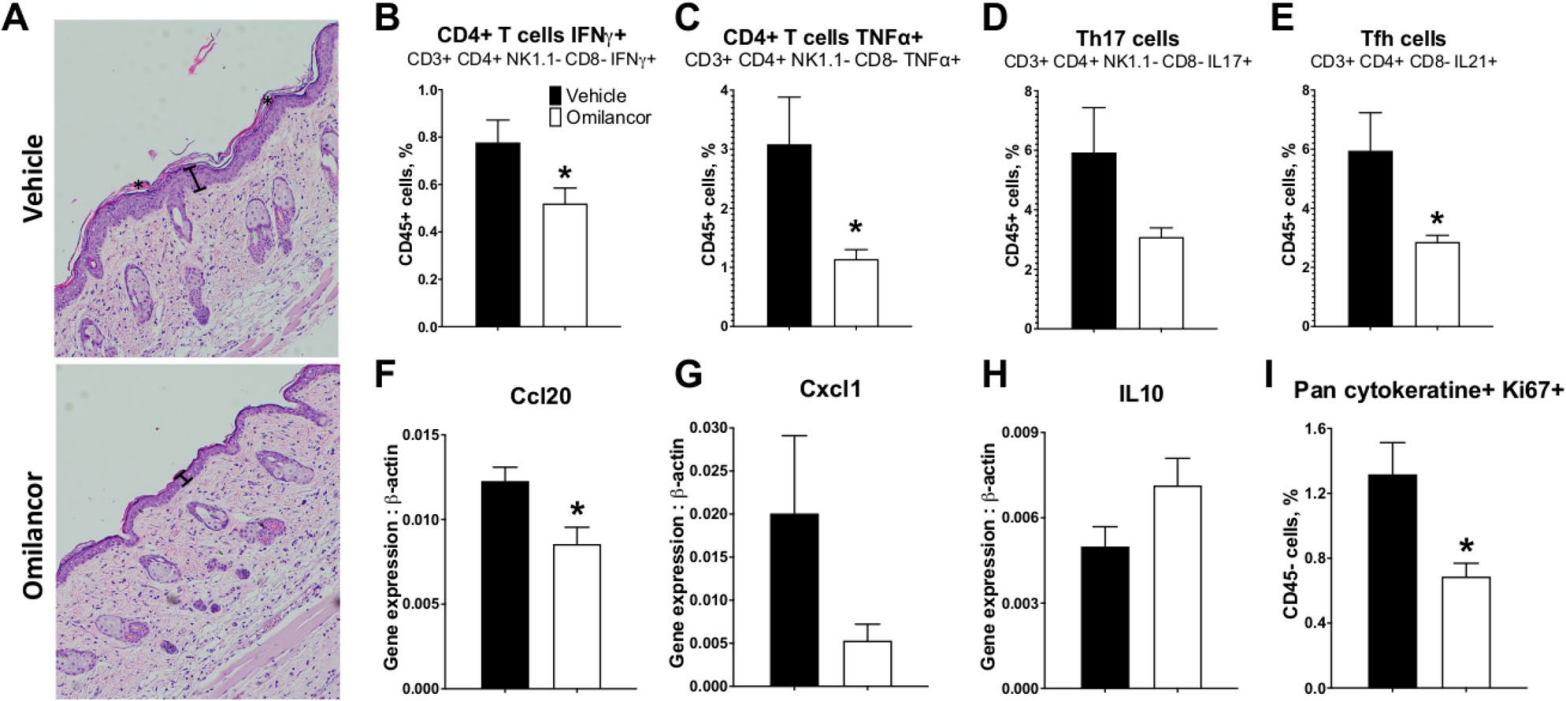
Therapeutic efficacy of topical omilancor treatment in IL-23-induced model of mouse PsO in dorsal skin. WT mice were shaved and daily challenged with intradermal injection of IL-23 at dorsal skin. Vehicle or omilancor formulations were administered daily 1 h post IL-23 injection. Mice were euthanized at day 4. Representative photomicrographs of H&E-stained dorsal skin at day 4 (**A**). Epidermal hyperplasia (|-|) and hyperkeratosis (*) are represented. Percentage of IFNγ+  CD4+ T cells (**B**), TNFα+ CD4+ T cells (**C**), Th17 cells (**D**) and Tfh cells (**E**) was quantified in draining ILNs through flow cytometry analysis. *Ccl20* (**F**), *Cxcl1* (**G**), and *IL10* (**H**) expression from dorsal skin was measured by qRT-PCR. Percentage of Pan cytokeratine+ Ki67+ cells (**I**) was assessed through flow cytometry analysis. *P ≤ 0.05.

The IL23/Th17 axis is a key player in the pathogenesis of PsO^21,22^. We sought to assess the therapeutic potential of omilancor upon activation of the IL23/Th17 pathway, and its effects on keratinocyte function, a critical component of PsO pathogenesis. WT mice were shaved as previously described. After 3 days, mice were daily intradermally injected with 1 µg of murine, recombinant IL-23 in their dorsal skin. Intradermal injections of IL-23 have been reported to induce a psoriasis-like phenotype including epidermal hyperplasia and dermal immune cell infiltration^49,50^. Omilancor treatment was applied topically 1 h post challenge. On day 4, mice were euthanized and ILN, spleen and dorsal skin collected for analysis. Our results indicate that this model induces very limited systemic inflammation (Supplementary Fig. S2), therefore we targeted the analysis locally. Skin LANCL2 activation through topical omilancor treatment reduced both epidermal hyperplasia and hyperkeratosis when compared to the untreated group (Fig. 3A). Flow cytometry analysis from draining ILNs, reported that omilancor-treated groups displayed a decrease in the recruitment of effector T cells subsets, including IFNγ positive cells (Fig. 3B), TNFα-producing T cells (Fig. 3C), Th17 cells (Fig. 3D) and follicular T cells (Fig. 3E). At the skin tissue level, omilancor treatment downregulated expression of *Ccl20* (Fig. 3F) and *Cxcl1* (Fig. 3G) and promoted expression of IL-10 (Fig. 3H). Further, activation of the LANCL2 pathway resulted in a decrease of keratinocyte proliferation, assessed by flow cytometry through expression of Ki-67 (Fig. 3I). These results support the therapeutic efficacy of omilancor in a second mouse model of PsO and elucidate the ability of topical omilancor treatment to modulate skin inflammation and keratinocyte proliferation. Therapeutic efficacy in this model suggests the potential role of the LANCL2 pathway in regulating the IL23/Th17 axis, as well as the subsequent keratinocyte activation.

### Lancl2 pathway activation down-regulates Th17 differentiation and function through immunometabolic mechanisms

**Figure 4:**
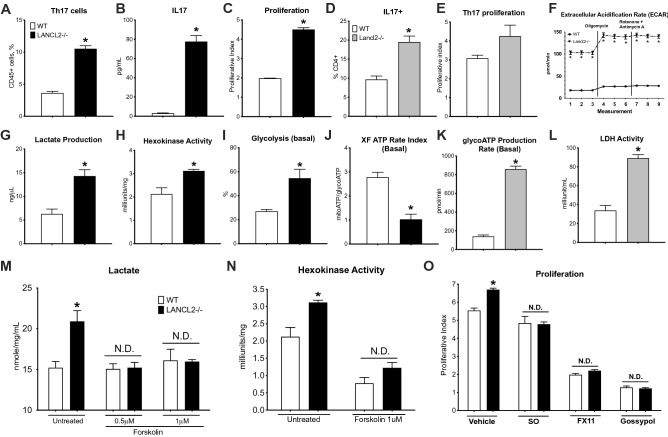
Effects of LANCL2 pathway in CD4+ T cells and Th17 cells. CD4+ T cells and naïve T cells from WT and LANCL2^−/−^ spleens were isolated and cultured for 48 h. Naïve T cells were differentiated to Th17 cells. In CD4+ T cells, proportion of Th17 cells (**A**) were measured using flow cytometry analysis, IL17 (**B**) secretion was quantified through cytokine bead array, and proliferation (**C**) through CFSE staining. In Th17 differentiated cells, percentage of IL17+ cells (**D**) and Th17 proliferation (**E**) were assessed through flow cytometry analysis. Metabolic assessment was also conducted using Agilent Seahorse (**F**,**I–K**) and commercial metabolic kits (**G**,**H**,**L–N**). Extracellular Acidification Rate (**F**), ATP production from glycolysis (**K**) and LDH activity (**L**) were assessed in Th17-differntiated cells. Lactate production (**G**), hexokinase enzymatic activity (**H**), percentage of utilization of complete glycolytic pathway (**I**) and XF ATP Rate Index (mitoATP/GlycoATP, **J**) were measured in CD4+ T cells. CD4+ T cells were treated with the adenylate cyclase activator Forskolin and lactate production (**M**) and hexokinase enzymatic activity (**N**) were measured using the metabolic kits. CD4+ T cells were also treated with sodium oxamate, FX11, and gossypol (three inhibitors of LDH activity) and proliferative index (**O**) was assessed by CFSE staining and flow cytometry analysis. *P ≤ 0.05.

The therapeutic efficacy upon administration of monoclonal antibodies targeting the IL23/Th17 axis in PsO validates the critical role of IL-17, and Th17 cells, in the pathogenesis of this condition^51,52^. Omilancor, and the LANCL2 pathway, are important regulators of CD4+ T cells. Indeed, omilancor promotes Treg differentiation and stability, and upregulates Treg suppressive capacity^34,35^. We sought to assess the role of LANCL2 pathway in modulating Th17 differentiation and function. CD4+ T cells were isolated and sorted from WT and Lancl2^−/−^ mice and cultured 48 h in anti-mouse CD3 pre-coated plates. In some experiments, naïve T cells were sorted from WT and Lancl2^−/−^ mice and differentiated into Th17 cells through exposure to a Th17-cytokine cocktail for 48 h. Lancl2 deficiency resulted in increased Th17 differentiation and proliferative capacity. In Lancl2-deficient CD4+ T cells, the percentage of Th17 cells (Fig. 4A) and the secretion of IL-17 (Fig. 4B) were upregulated. Lancl2^−/−^ Th17-differentiated cells also displayed increased expression of IL-17 compared to the WT group, as measured by flow cytometry (Fig. 4D**)**. Further, proliferative assessment through CFSE staining indicated that loss of Lancl2 in CD4+ T cells and differentiated Th17 cells results in higher proliferative index (Fig. 4C,E).

The metabolic reprogramming of CD4+ T cells is tightly associated to their specific function. Indeed, Regulatory and effector T cells display very distinctive metabolic profiles. To successfully conduct effector responses and meet the increased energy demands, effector T cells undergo a critical metabolic switch, characterized by an increase in glycolysis rate and lactate production^53,54^. Thus, modulation of metabolism in Th17 cells leverages a new mechanism to regulate Th17 function. Lancl2^−/−^ cells display upregulated glycolytic rates and anaerobic metabolism. In CD4+ T cells, both lactate production (Fig. 4G) and hexokinase activity (Fig. 4H) are upregulated due to the loss of Lancl2. Additionally, Seahorse analysis reported increased glycolysis (Fig. 4I) as well as XF ATP Rate index (Fig. 4J), ratio between ATP production from oxidative phosphorylation versus ATP from glycolysis, in Lancl2^−/−^ cells. In accordance with the CD4+ T cells results, metabolic assessment of Th17-differentiated cells also indicated elevated glycolytic rate and lactate production due to the loss of Lancl2. This was observed as upregulated ECAR (Fig. 4F), ATP production from glycolysis (Fig. 4K), and lactate dehydrogenase activity (LDH, Fig. 4L) in Lancl2^−/−^ cells.

To validate the LANCL2 dependency of the observed metabolic changes, CD4+ T cells were treated with the adenylate cyclase activator forskolin. Interestingly, downstream activation of the LANCL2 pathway through forskolin treatment abrogates the increased lactate production (Fig. 4M) and hexokinase activity (Fig. 4N) reported in Lancl2 deficient cells. Additionally, inhibition of a key metabolic enzyme of glycolysis, LDH, through treatment with the LDH inhibitors (sodium oxamate, FX11 and gossypol) abrogates the differences in proliferative rate reported between WT and LANCL2^−/−^ cells (Fig. 4O). This indicates that the Lancl2 pathway is involved in the regulation of Th17 effector function and proliferative activity, and it involves the modulation of glucose metabolism, at early and late stages.

### In vivo omilancor treatment modulates the metabolic profile of psoriatic keratinocytes

**Figure 5:**
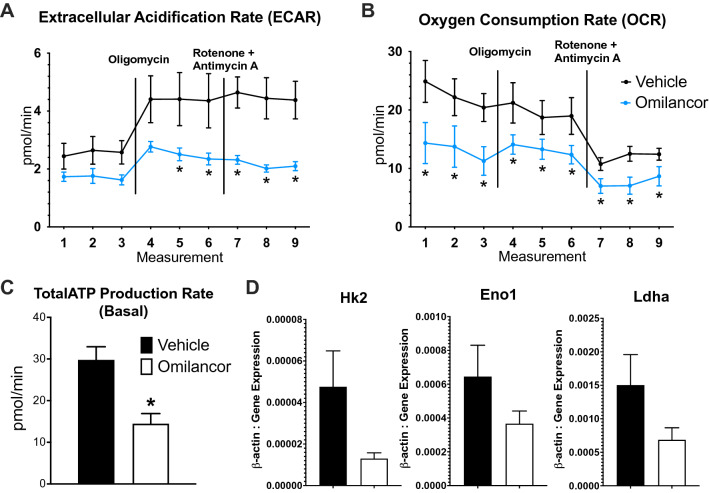
Metabolic effects of in vivo topical omilancor treatment in psoriatic keratinocytes. KC from vehicle- and omilancor-treated mice were isolated at day 7 post IMQ-challenge. ECAR (**A**), OCR (**B**) and total ATP production (**C**) were assessed using Agilent Seahorse instrument. *Hk2*, Eno1 and *Ldha* expression was quantified by qRT-PCR in dorsal skin (**D**). *P ≤ 0.05.

In psoriatic lesional skin, keratinocytes display hyperproliferation, altered differentiation and upregulated production of cytokines and chemokines. Similar to what has been reported in effector T cells, psoriatic keratinocytes undergo a metabolic reprogramming to quickly meet the increased energy demands to supply the upregulated proliferation rate and protein signaling^55^. Indeed, psoriatic keratinocytes isolated from dorsal skin of a 7-day IMQ-treated mice, displayed increased ECAR (Supplementary Fig. S3B), oxygen consumption rate (OCR), parameter directly associated to mitochondrial respiration (Supplementary Fig. S3A), and overall ATP production (Supplementary Fig. S3C) compared to keratinocytes of non-inflamed skin. Based on the metabolic implication of the LANCL2 pathway in modulating metabolism of CD4+ T cells, and other non-immune cells^34,35,37^, here we assessed the potential effects of topical omilancor in keratinocyte metabolism. Using the IMQ-induced model of skin inflammation, psoriatic keratinocytes were isolated from dorsal skin of WT mice topically treated with vehicle or omilancor for 7 days. The metabolic profile from isolated psoriatic keratinocytes was assessed using Agilent seahorse technology. Our results indicate that in vivo omilancor treatment downregulates both ECAR (Fig. 5A) and OCR (Fig. 5B) in psoriatic keratinocytes. Additionally, total ATP production was also reduced in the omilancor-treated group (Fig. 5C). Further, gene expression analysis also indicated a downregulation of several glycolytic genes, *hexokinase*, *enolase* and *ldha* (Fig. 5D) associated to activation of LANCL2 pathway in dorsal skin compared to the untreated group. This indicates that topical omilancor treatment correlates with a distinctive metabolic profile in inflamed skin cells, characterized by increased energy production due to significant upregulation of both glycolytic rate and mitochondrial metabolism in psoriatic keratinocytes. Thus, topical omilancor prevents the dysregulated metabolic reprogramming observed in psoriatic keratinocytes, and induces a phenotype resembling the metabolic profile of keratinocytes isolated from non-inflamed tissue.

## Discussion

This study supports the development of a new topical omilancor formulation as a novel, first-in-class, therapeutic for the treatment of PsO and other skin conditions. Daily topical omilancor treatment provides therapeutic efficacy in two mouse models of psoriasis through downregulation of inflammatory responses and metabolic switches. Even though omilancor is a locally acting molecule with very low systemic exposure, and a plasma half-life of 3.08 h when administered orally^56^, topical administration in the skin provides over 50% reduction of inflammation in PsO within the lymph nodes and spleen, suggesting that the systemic inflammation in these models is driven by the local immune environment. Locally, we report protective actions of omilancor at two levels: immune cells and keratinocytes. Indeed, LANCL2 is expressed in immune cells and keratinocytes. Furthermore, the activation of LANCL2 by omilancor induces a decreased infiltration of proinflammatory immune cell types, including CD4+ T cells, macrophages and neutrophils, while also abrogating excessive keratinocyte activation. As a result, topical omilancor treatment reduces the severity of skin lesions, and promotes the preservation of healthy skin composition and structure.

To assess the therapeutic efficacy of topical activation of LANCL2 in PsO, we utilized two distinctive murine models of PsO-like inflammation. The IMQ topical challenge was initially selected as it is a widely utilized model that resembles human PsO and induces skin inflammation through an IL-23/Th17-dependent mechanism^47^. To confirm the generated results, we utilized the IL-23 intradermal injection model. Intradermal injections of IL-23 induce epidermal hyperplasia, erythema and infiltration of immune cells^50^. Even though it was initially postulated that skin inflammation induced by intradermal IL-23 injections is not dependent of IL-17A signaling^50^, recent loss-of-function studies have demonstrated that IL-17A is required to develop skin inflammation upon IL-23 intradermal injections^49^.

The IL-23/Th17 axis has been established as one of the main players in the pathogenesis of PsO. Immunophenotyping analysis in vivo reported a consistent decrease of IL-17 production by CD4+ T cells associated to topical omilancor administration both in spleen and local LN. In vitro, loss of Lancl2 in CD4+ T cells resulted in dysregulated Th17 differentiation and function, with increased IL-17 production and cell proliferation. PsO patients displayed an imbalanced Th17/Treg ratio, and Treg cells present impaired suppressive function^25,57^. Previously, we have identified the mechanisms by which LANCL2 activation regulates Treg stability and function^34,35^. Together, these data suggest that the therapeutic efficacy resulted from pharmacological activation of Lancl2 by omilancor in PsO might be dependent on the modulation of Th17 responses. Commercial biologic therapeutics that target the IL23/Th17 axis through administration of systemic monoclonal antibodies (anti-IL17A, and -IL23), have transformed the treatment paradigm of PsO due to their high efficacy rates^51,58,59^. However, the non-discriminated suppression of the targeted signaling pathway by the blocking of monoclonal antibodies can lead to the development of important side effects, such as the development of severe opportunistic infections or certain types of cancer. Activation of the LANCL2 pathway by omilancor regulates Th17 responses, without inducing systemic immune suppression. Thus, omilancor has the potential to meet and exceed the efficacy of current biological therapies without the risk of developing adverse side effects, as observed in both Phase 1 and 2 omilancor clinical trials in UC patients. Additionally, the ability of omilancor to induce strong Treg responses in the skin could induce and maintain longer remission periods. Regarding the route of administration, omilancor treatment as a topical formulation eliminates the need to use injectable, as current biologics do, leading to a much more convenient administration for patients. In addition to regulate the immunological compartment, omilancor treatment also modulates epidermal keratinocyte activation. LANCL2 activation in the skin through topical omilancor treatment suppresses keratinocyte proliferation and expression of proinflammatory markers, resulting in reduced epidermal hyperplasia as well as infiltration of immune cells, as observed in our histological analysis of skin samples from mice with PsO. Local IL-17 and IL-22 in psoriatic skin are the main drivers of keratinocyte activation. Indeed, they activate epidermal keratinocytes, promoting hyperproliferation, production of antimicrobial peptides and pro-inflammatory markers^21,30^. A proposed mechanism of action of topical omilancor treatment in PsO is that activation of LANCL2 pathway down-regulates Th17 activation, reducing the production of proinflammatory cytokines, that then leads to a decreased skin influx of proinflammatory immune subsets, i.e. neutrophils and proinflammatory macrophages, and suppressed keratinocyte activation. Consequently, omilancor topical treatment ameliorates disease severity, reporting higher preservation of skin structure, with lower epidermal hyperplasia and acanthosis. Additionally, the high Lancl2 expression levels in epithelial cells, together with the fact that omilancor treatment modulates similar cellular functions in CD4+ T cells and keratinocytes (proliferation, metabolic profile and expression of proinflammatory markers), suggest that omilancor treatment might also modulate keratinocyte activation through direct activation of LANCL2 in keratinocytes. Further in vitro studies will be conducted to confirm this hypothesis.

In this study we also investigated the potential of LANCL2 signaling in modulating immunometabolic pathways during PsO and its therapeutic consequences. The complex and dynamic crosstalk between cellular immunity and metabolism leverages the development of novel therapeutic strategies that induce regulatory responses at the intersection of cellular metabolism and immune cell function. Upon the initiation of the immune response, activated immune cells undergo a metabolic switch to meet the energy and biomass requirements to successfully execute effector mechanisms. To generate energy (ATP) at high speed, effector cells leverage an anaerobic metabolism characterized by increased glucose consumption and lactate production, while in regulatory cells, oxidative mitochondrial metabolism is favored, presenting increased complete glucose catabolism through TCA cycle and oxidative phosphorylation as well as fatty acid oxidation^53,54,60–62^. Activation of the Lancl2 pathway results in the induction of immunometabolic mechanisms that enhance Treg differentiation and function by promoting Foxp3 stability and immune checkpoint expression^34,35^. In effector CD4+ T cells, and Th17 cells, the lancl2 pathway regulates anaerobic metabolism, both at early and late stages of glycolysis. Loss of Lancl2 results in upregulated glycolytic rates and increased production of ATP from glycolytic origin. Th17 cells depend more on the glycolysis in comparison to other cell types^63,64^. It has been previously reported that lactate induces upregulation of *Il17* and *rorc* expression, promoting the generation of Th17 cells^65^. Our data indicates that the LANCL2 pathway modulates glycolytic rates through regulation of Hexokinase, PDH^35^ and LDH activity. Hexokinase controls the first glycolytic reaction, that constitutes the first-rate limiting step. LDH is responsible for the last glycolytic step, that converts pyruvate in lactate through production of NAD+. LANCL2-dependent downregulation of the activity of these two enzymes reduces flux through the entire glycolytic pathway and regulates the activation of Th17 cells. Importantly, the inhibition of LDH activity abrogated the increased proliferative rate in CD4+ T cells associated to loss of Lancl2. Omilancor promotes PDH activity in Treg cells, facilitating the mitochondrial use of pyruvate^35^. These suggest a double mechanism of the LANCL2 pathway regulating Th17 generation through downregulation of glycolytic rate in effector T cells, (1) downregulating the activity of two key glycolytic enzymes (HK, LDH), that limit glycolysis rate and present association to proliferation, and (2) enhancing pyruvate entrance to TCA cycle through increased PDH function.

Our results also indicate a decrease in keratinocyte metabolic activity upon topical omilancor treatment. The increased proliferative rate and turnover observed in psoriatic keratinocytes require the induction of metabolic changes to quickly increase energy production and substrate availability for de novo synthesis of biomolecules that will support the high dividing cell rate. Indeed, proliferating keratinocytes display upregulated expression of the metabolite transporters GLUT1 (glucose), LAT1 and CAT1 (amino acids)^66^. Proliferative keratinocytes depend on glucose metabolism, since deficiency of glut1 decreases keratinocyte proliferation^66^. The LANCL2 pathway modulates the metabolic reprogramming of epidermal keratinocytes, and abrogates the increase in glycolytic rate, but also the upregulation in oxidative phosphorylation. Glut1 expression is upregulated in psoriatic lesional skin of patients^66^. In a mouse model of PsO, inhibition of Glut1 results in decreased keratinocyte proliferation and epidermal hyperplasia in PsO^66^. This suggests that keratinocyte high proliferative rate and epidermal hyperplasia observed in PsO is dependent on the keratinocyte metabolic reprogramming. Therefore, through modulation of metabolic upregulation in keratinocytes, omilancor treatment suppresses keratinocyte proliferation, accelerated turnover and thickening of the skin, resulting in lower disease severity.

In conclusion, this manuscript reports the therapeutic efficacy of topical omilancor administration in the treatment of PsO in two mouse models. Our results indicate that pharmacological activation of the LANCL2 pathway through omilancor downregulates the activation of Th17 cells and epidermal keratinocytes through activation of regulatory mechanisms at the interface of immunity and metabolism. More specifically, the activation of the LANCL2 pathway by omilancor regulates glucose metabolism and lactate production, leading to the down-modulation of key cell functions in both immune and epidermal skin cells, including production of proinflammatory cytokines and chemokines as well as regulation of cell proliferation. Thus, topical omilancor treatment has the potential to restore the tissue homeostasis that is lost in psoriatic lesions skin. These data, not only support the development of omilancor for the treatment of psoriasis, but also leverage the exploration of a new class of LANCL2-based therapeutics that target immunomodulatory mechanisms for the treatment of PsO and other inflammatory skin disorders.

## Materials and methods

### Animal housing and ethic statement

C57Bl/6 wild-type (WT) and LANCL2 whole body knock out (Lancl2^−/−^) mice were housed and bred in the same colony, in ventilated racks, and a 12:12 lighting cycle. Mice had water and food at libitum. Mice were humanely euthanized through CO_2_ narcosis followed by cervical dislocation as a secondary method. All experimental procedures performed were approved by the Biotherapeutics Institutional Animal Care and Use Committee (IACUC), met or exceeded requirements of the Public Health Service/National Institutes of Health and Animal Welfare Act and were conducted according to the approved guidelines and regulations. This study is reported in accordance with ARRIVE guidelines.

### Mouse models of psoriasis (PsO)

8 to 11-week-old C57Bl/6 WT mice were anesthetized with isoflurane, the dorsal back skin was shaved, and depilatory cream was briefly applied. After 3 days, mice were challenged. For the imiquimod (IMQ)-induced model, mice were challenged daily with 3.125 mg of IMQ topically on the shaved area for 7 days. Mice were monitored daily and scored for skin erythema, thickness and scaling. Note, PASI was represented as the score sum of the three monitored parameters. For the IL23-induced model of PsO, mice were administered 1 µg of murine recombinant IL-23 (BioLegend) intradermally in two sides of the shaved dorsal back daily for 4 days. For the IMQ-induced model in mouse skin ears, mice were administered 0.372 mg of IMQ daily in both sides of each year for 7 days. For omilancor efficacy studies, mice were topically administered omilancor in the form of a 2% (w/w) omilancor cream. Mice were administered 5 mg of 2% (w/w) cream per cm^2^ of skin treated (0.1 mg of omilancor/cm^2^) once daily at 1 h post challenge. At the end of the challenge, mice were euthanized for collection of tissues for downstream assays.

### In vitro CD4+ T cell isolation, differentiation and culture

Spleens from C57Bl/6 wild-type (WT) and Lancl2^−/−^ mice were excised, crushed and red blood cells lysed. CD4+ T cells were negatively sorted using the BD Biosciences enrichment cocktail following manufacturer’s instructions. For naïve T cell isolation, CD4+ T cells were incubated with biotin conjugated anti-mouse CD62L antibody for 15 min, followed by 30 min of incubation with the BD IMag Streptavidin Particles Plus—DM. Then samples were incubated in a magnet and labelled naïve T cells were positively sorted. 200,000 CD4+ T cells or naïve T cells were plated in round-bottom 96-well plates pre-coated with anti-mouse CD3 (BD Biosciences) and cultured for 48 h. For Th17 differentiation, naïve T cells were supplemented with a Th17-differentiation cytokine cocktail containing murine recombinant IL-6, IL-23, TGFb (PeproTech), anti-mouse IL-4 and anti-mouse IFNγ (BD Biosciences). In some specific experiments, CD4+ T cells cells were also incubated with the adenylate cyclase activator forskolin, and the lactate dehydrogenase inhibitors sodium oxamate, FX-11, and gossypol during the 48-h culture. 6 h prior harvesting, CD4+ T cells and Th17 differentiation cells were stimulated with Ionomycin (500 ng/mL) and PMA (5 ng/mL). Samples for flow cytometry analysis were also incubated with a protein transport inhibitor (BD Biosciences) to prevent secretion of intracellular cytokines. To assess proliferation, cells were stained with CFSE.

### Histopathology

Dorsal skin samples were fixed in 10% formalin, paraffin-embedded, sectioned and Hematoxylin and eosin (H&E) stained. Slides were examined and graded using an Olympus microscope in a scale from 0 to 4 for inflammatory cell infiltrate, epidermal hyperplasia, and hyperkeratosis.

### Flow cytometry and cytokine bead array

Spleen, inguinal lymph nodes (ILN), cervical lymph nodes (CLN) and dorsal skin were excised. Tissues were digested to obtain single-cell suspension. Spleens were crushed and red blood cells lysed. ILN and CLN were incubated 1 h at 37 °C stirring in RPMI media supplemented with Collagenase and DNase. For dorsal skin samples, epidermal cells were isolated as described in Ref.^67^. Briefly, dorsal skin was excised, and subcutaneous tissue was scraped using a scalpel blade. Skin samples were then incubated 2 h at 32 °C in trypsin, epidermal side up. Epidermal cells were scraped off using a scalpel blade and incubated 20 min stirring. Single cell suspensions from tissues and CD4+ T cells and differentiated Th17 cells were plated in 96-well plates for immunophenotype analysis. Samples were incubated in Fc block, followed by a mixture of fluorochrome-conjugated antibodies. Samples were then fixed and permeabilized (Invitrogen) and stained with a mixture of fluorochrome-conjugated antibodies against transcription factors and cytokines. Fluorochrome-conjugated antibodies utilized included: anti-CD45, CD3, CD4, CD8, NK1.1, CD25, CD11b, Gr1, TNFα, IFNγ, IL17, RORγt, IL21, IL-10, Foxp3, Pancytokeratin, ki67 and IL6. Samples were acquired using a BD FACSCelesta instrument and analyzed using BD FACSDiva software (BD Biosciences).

To assess IL-17 protein levels, supernatants from CD4+ and Th17-differentiated cells were collected and frozen and − 80 °C. Secreted IL-17 was assessed using the cytokine bead array (BD biosciences) following manufacturer’s instructions.

### Gene expression

Dorsal skin samples were excised and stored in RNAlater at − 80 °C. Total RNA was extracted using the RNeasy kit (QIAGEN) following manufacturer’s instructions. cDNA was generated using the iScript cDNA synthesis kit (Bio-Rad). Purified primer-specific amplicons were used to generate standard curves for each gene analyzed. Total gene expression was quantified through qRT-PCR using Sybr Green mix (Bio-Rad) and a CFX96 Thermal cycle. Gene expression levels are expressed targe genes normalized to beta-actin expression.

### Metabolic analysis

Extracellular acidification rate (ECAR) and oxygen consumption rate (OCR) were measured using an Agilent Seahorse Xf96e instrument. Cells were plated in a 96-well seahorse plate and incubated 1 h at 37 °C in a non-CO_2_ incubator with Seahorse media supplemented with 1 mM glucose, pyruvate and glutamine. The ATP Real time assay kit, that includes an initial injection of 1.5 µM of oligomycin, followed by 0.5 µM of Rotenone and Antimycin AA was utilized. This assay allowed the calculation of several metabolic parameters regarding ATP production and metabolic profile, including total ATP, GlycoATP, % of glycolysis and XF ATP Rate Index.

Commercial metabolic kits were utilized to assess lactate production, hexokinase activity, and lactate dehydrogenase activity. Briefly, cells were homogenized in assay buffer, centrifuged and supernatant containing cell lysates used for the analysis following manufacturer’s instructions.

### Statistical analysis

Analysis of variance (ANOVA) was carried in R to determine significance of the data. Significance was identified with an asterisk (*) and considered at *P*-value ≤ 0.05. Data is expressed as the mean and standard error of the mean represented in error bars.

## Supplementary Information

Supplementary Figures.
