# Supplementary material for: First-in-class topical therapeutic omilancor ameliorates disease severity and inflammation through activation of LANCL2 pathway in psoriasis
Source: Sci Rep. 2021 Oct 6;11:19827. doi: 10.1038/s41598-021-99349-y (PMC8494925; doi:10.1038/s41598-021-99349-y)
Supplement: Supplementary file 1 — Supplementary Figures. [file 41598_2021_99349_MOESM1_ESM.pdf]

# First-in-class topical therapeutic omilancor ameliorates disease severity and inflammation through activation of LANCL2 pathway in psoriasis

Nuria Tubau-Juni[1], Raquel Hontecillas[1], Andrew Leber[1], Panita Maturavongsadit[1], Jyoti Chauhan[1], and Josep Bassaganya-Riera[1]

[1] Landos Biopharma, Inc., Blacksburg, VA 24060, USA.

**Correspondence:** Dr. Josep Bassaganya-Riera, Landos Biopharma, Inc. (www.landosbiopharma.com), Blacksburg, VA, 24060, USA. E-mail: jbr@landosbiopharma.com; phone: (540) 218-2232.

## Supplementary information

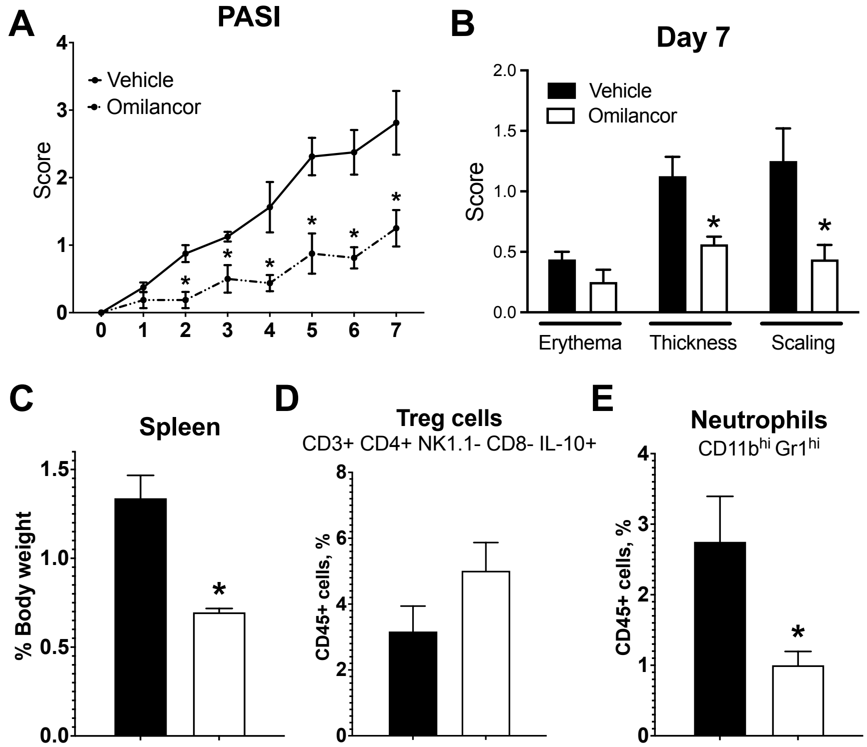


**Supplementary Figure S1. Efficacy of topical omilancor treatment in an IMQ-induced model of PsO in ear skin.** Ear skin of WT mice were challenged with IMQ for 7 d. Vehicle or omilancor topical formulation was applied daily 1 h post IMQ challenge. Disease activity was monitored daily using a modified PASI (**A**). Skin erythema, thickness, and scaling (**B**), at day 7 post challenge. Spleen size (**C**) represented as proportion of total body weight. Percentage of Treg cells (**D**) and Neutrophils (**E**) were quantified by flow cytometry analysis. * $P \leq 0.05$.

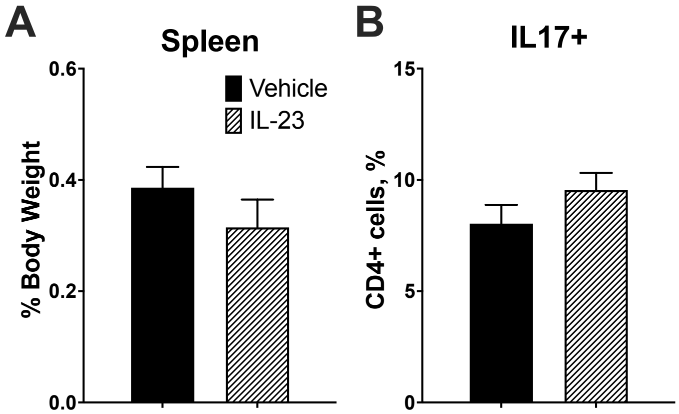


**Supplementary Figure S2. Limited systemic effect of IL-23-induced model of PsO.** WT mice were shaved and daily challenged with IL-23 or vehicle through intradermal injection at dorsal skin. Spleen size (**A**) represented as proportion of total body weight. Percentage of IL17+ cells (**B**) in CD4+ T cells was assessed in the spleen by flow cytometry analysis. Ear skin of WT mice were challenged with IMQ for 7 d.

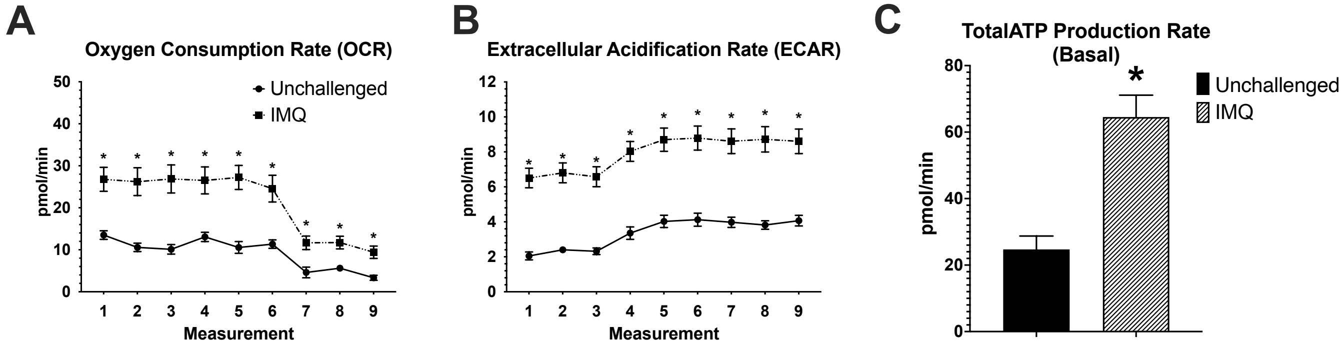


**Supplementary Figure S3. Assessment of metabolic profile in IMQ-challenged keratinocytes in comparison to unchallenged controls.** WT mice were shaved, and challenged with IMQ during 7 d. An unchallenged control group was also included. Psoriatic and non-inflamed keratinocytes were isolated at d 7 post challenge. OCR **(A)**, ECAR **(B)** and total ATP production **(C)** were measured using Agilent Seahorse. * $P \leq 0.05$.

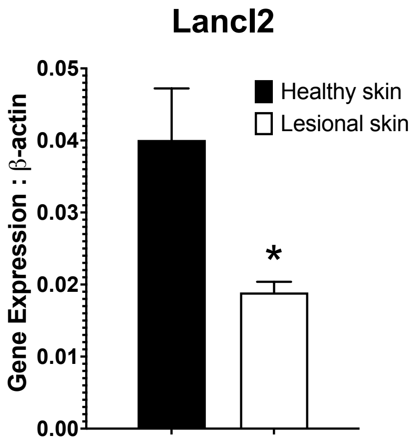


**Supplementary Figure S4. Assessment of lancl2 expression in skin of IMQ-challenged mice in comparison to unchallenged controls.** WT mice were shaved, and challenged with IMQ during 7 d. An unchallenged control group was also included. RNA from healthy and psoriatic skin was isolated at d 7 post challenge. Lancl2 expression was assessed through qRT-PCR. * $P \leq 0.05$.
